# Origin and Evolution of Deleterious Mutations in Horses

**DOI:** 10.3390/genes10090649

**Published:** 2019-08-28

**Authors:** Ludovic Orlando, Pablo Librado

**Affiliations:** 1 Laboratoire d’Anthropobiologie Moléculaire et d’Imagerie de Synthèse, CNRS UMR 5288, Université de Toulouse, Université Paul Sabatier, 31000 Toulouse, France; 2 Globe Institute, Faculty of Health and Medical Sciences, University of Copenhagen, 1350K Copenhagen, Denmark

**Keywords:** horse, genomics, deleterious variants, mutational loads, negative selection

## Abstract

Domestication has changed the natural evolutionary trajectory of horses by favoring the reproduction of a limited number of animals showing traits of interest. Reduced breeding stocks hampered the elimination of deleterious variants by means of negative selection, ultimately inflating mutational loads. However, ancient genomics revealed that mutational loads remained steady during most of the domestication history until a sudden burst took place some 250 years ago. To identify the factors underlying this trajectory, we gather an extensive dataset consisting of 175 modern and 153 ancient genomes previously published, and carry out the most comprehensive characterization of deleterious mutations in horses. We confirm that deleterious variants segregated at low frequencies during the last 3500 years, and only spread and incremented their occurrence in the homozygous state during modern times, owing to inbreeding. This independently happened in multiple breeds, following both the development of closed studs and purebred lines, and the deprecation of horsepower in the 20th century, which brought many draft breeds close to extinction. Our work illustrates the paradoxical effect of some conservation and improvement programs, which reduced the overall genomic fitness and viability.

## 1. Introduction

The domestication of the horse deeply impacted human history, enhancing the mobility of people, trade, and culture. For example, the diffusion of Indo-European languages has been associated with migration waves of horseback riders [1,2]. Following the incorporation of chariotry and cavalry into warfare, domestic horses have played a crucial role in the rise and fall of entire past civilizations. It was only with the onset of motor vehicles in the early 20th century that the horse remained consigned to farming and transportation in developing countries, and to recreation in Westernized societies. The equine industry remains instrumental today, with a census population size of 58.5 million horses, and a yearly market worth 300 billion US dollars [3].

Breeders have reshaped the horse evolutionary trajectory by controlling its reproduction for hundreds of generations [4]. Artificial selection has engendered several hundred breeds, showing striking phenotypic differences in a range of traits, including morphology, coat coloration, working capacities, and speed. However, developing such a broad array of breed-associated phenotypes has entailed profound genomic changes as demonstrated by Fages et al., who recently generated an extensive genome time-series spanning the last five millennia [5]. The authors found heterozygosity levels remaining relatively steady until they dropped by ~16% some 200 years ago. This clearly revealed that modern reproductive strategies have considerably reduced horse genetic diversity, possibly through the incorporation of only a limited number of influential stallions [5,6,7].

Reducing the size of the breeding stock holds the potential to also limit the power of negative selection against deleterious mutations. Consistent with this prediction, Fages et al. estimated that horses that lived after the 18th century evolved under weaker selection, which led to a ~4% increment in homozygous deleterious variants within their protein-coding regions [5]. Although convincingly confirming that modern practices were responsible for the mutational burst, this analysis only included 23 present-day horses. This number was not sufficient to contrast the multiple breeding practices implemented in modern times, and their potential impact on shaping present-day loads.

Understanding such causes has important implications for the equine industry, as deleterious variants can reduce the individual fitness and viability, even in the heterozygous state [8,9]. Developing monitoring tools for harmful variants could improve the sustainability of breeding practices. However, the list of known deleterious variants is extremely limited, with only 90 causative variants catalogued thus far in the OMIA database (Online Mendelian Inheritance in Animals) [10]. Genome-Wide Association Studies (GWAS) are indeed often underpowered for deleterious variant discovery, due to the complex architecture of the phenotypic traits investigated and the underlying pleiotropic interactions masking the exact contribution of each individual allele [11]. Therefore, one of the most feasible approaches to identify sustainable breeding practices, ensuring the long-term viability of breeds, is to investigate their impact on the accumulation of deleterious mutations [12,13,14].

The relation between breeding strategies and mutational burdens can be more complex than simply limiting the efficacy of natural selection in filtering out deleterious mutations. The most obvious mechanism decoupling the expected positive relation between inbreeding and load is genetic purging [15]. In this process, recessive mutations that were hidden in the heterozygous state are brought to the homozygous state through inbreeding, and thus phenotypically exposed to negative selection. Whether genetic purging and other mechanisms have contributed to shape present-day mutational loads also remains to be elucidated.

In this study, we performed the most extensive characterization of deleterious mutations in horses. We collected the genome sequences of 161 horses from 36 domestic breeds, 14 Przewalski horses, as well as one domestic donkey, all sequenced to an average depth-of-coverage of 5–39 ×. We extended the genomic load estimator applied in previous studies to also cover non-coding regions, and leveraged the extensive data from Fages et al. [5] to investigate the temporal origins and evolutionary trajectories of deleterious variants. This work provides new insights into the genomic impact of recent reproductive breeding and conservation techniques.

## 2. Materials and Methods

Sequencing data from modern horses were retrieved from ENA and NCBI repositories. Raw reads were processed and mapped against EquCab2 using PALEOMIX with default parameters [16]. Horse IDs, breed assignments, accession codes, and the resulting average depth-of-coverage per genome are reported in Table S1.

Evolutionary conservation is known to represent an excellent predictor of fitness effects, because mutations at sites that remained highly constrained during evolution are likely to be deleterious. We thus used phyloP conservation scores to estimate the potential impact of mutations. These scores summarize the evolutionary constraint position-wise, across a genome-wide alignment of 46 vertebrate species [17,18], including EquCab2 as reference assembly for the horse genome [19]. We considered as harmful all mutations at positions showing a minimum phyloP score of 1.5, which is a threshold that accurately discriminates fourfold and zerofold degenerate sites [20], i.e. synonymous (nearly-neutral) and non-synonymous (functional) variants, respectively. To calculate mutational loads per individual genome, we first estimated the genotype probabilities at each site, using ANGSD v0.917 [21] with the GATK likelihood model (-GL 2), and the following filtering parameters: -Uniqueonly 1 -remove_bads 1 -C 50 -baq 1 -minMapQ 30 -minQ 30 -only_proper_pairs 1. The most likely genotype was called and considered further, provided that its likelihood exceeded 0.99 and that it was homozygous (the phenotypic effect of deleterious variants at heterozygous sites depends on their unknown recessive, dominant, or co-dominant mode of inheritance). Sites were masked otherwise. Given both phyloP scores and genotype calls, we estimated the genetic load for each horse genome as:

$$load=\frac{\sum_{i}phyloP_{i}\;}{\#homozygous}$$

where *i* iterates over all the homozygous positions carrying a deleterious allele, and *phyloP_i_* is the phyloP score at the genomic position *i*. We assigned as deleterious the less frequent (or absent) allele in the 46-way alignment, provided that at most two variants were segregated.

Two approaches were applied to characterize the historical periods yielding inflated mutational loads. First, we retrieved all the previously published and radiocarbon-dated ancient genomes from ENA (PRJEB31613). After excluding mules, donkeys, and specimens belonging to other non-caballine lineages (i.e., not ancestral to modern horse breeds), we retained genomic data from 153 ancient horses (Table S2). These mostly lived during the last 3500 years. Then, we binned them within temporal windows of 1000 years, sliding every 250 years. For each time period, we estimated the frequency *f* of each deleterious allele from read count data using Maximum Likelihood (ML), where:

$$f=argmax_{p}\;\overset{n\_ind\_t}{\underset{i=1}{\prod }}Binomial\left(r_{di},\;d_{i},\;p\right)$$

where *n_ind_t* is the number of samples within each time interval, *d_i_* is the sequencing depth at that given position, and *r_di_* is the number of reads supporting the deleterious allele in individual *i*. We only considered those time bins showing at least 10 ancient horses genotyped, to minimize the variance associated with the estimation of *f*. By applying the same approach to the joint panel of 161 present-day domesticates (Table S1), we finally reconstructed the full temporal trajectory of each individual deleterious variant. Analyses were repeated considering transversions only, to limit the impact of post-mortem DNA misincorporations [22].

As a second approach to identify time periods of increasing loads, we calculated pairwise genetic distances with plink v2 [23] from a matrix that included the 161 modern domesticates, 14 Przewalski horses, and a donkey. We conditioned on 1,839,707,069 neutral positions (phyloP < 1.5), with one missing genotype at most. From these pairwise distances, we constructed a neighbor-joining (NJ) tree with subsequent topology refinement (*-n* option) [24]. Since low sequencing depths distort phylogenetic distances, all the genomes were pseudo-haploidized following a standard procedure in ancient DNA research. This consists in the random selection of one high-quality read at a given site as representative for the homozygous genotype. The tree branch lengths were used as proxies for neutral substitution rates, potentially revealing past episodes of elevated drift such as demographic collapses.

Pseudo-haploidized data were also used to characterize genetic purging, which involves selection against recessive mutations that are phenotypically exposed, such as those found at the homozygous state, both within and without Runs of Homozygosity (ROHs) resulting from inbreeding [25]. We approximated the strength selection by calculating the average genetic divergence of one given horse individual to the domestic donkey at constrained (dN; phyloP > 1.5) and neutral sites (dS; phyloP < 1.5). Strong negative selection purges out mutations at constrained sites, reducing dN and leading to negative dN˗dS values. Conversely, deleterious variants are not efficiently removed under relaxed negative selection. Thus, dN behaves more neutrally, and effectively approaches dS, leading to dN˗dS values closer to 0.

Finally, we estimated inbreeding coefficients, proceeding independently for Przewalski horses and modern domesticates. Specifically and for each group, we calculated the genotype posterior probabilities with ANGSD [21], and retained sites showing at most 10% missingness, provided that they segregated in approximate linkage equilibrium. The latter condition was satisfied through LD pruning and the calculation of *r*^2^ [26] for Single Nucleotide Polymorphism (SNP) pairs located less than 50 Kb away in ngsLD [27]. We next clustered these SNP pairs into larger groups of linked variants using mcl [28], and selected the most central SNP as representative of each block. This yielded a total of 1,249,153 and 6,244,327 high-quality SNPs for Przewalski horses and modern domesticates, respectively. Inbreeding coefficients and IBD tracts were then co-estimated on these sites applying ngsF-HMM with a strict convergence criterion (min-epsilon = 1e^−7^) [29].

## 3. Results

### 3.1. Levels and Patterns of Mutational Loads, Across Site Categories and Breed Types

**Figure 1:**
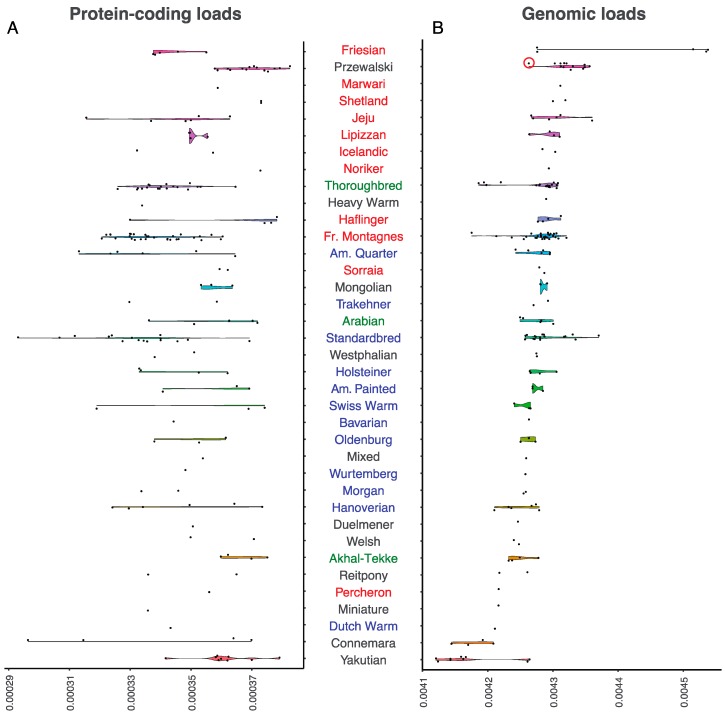
Mutational loads per breed, based on positions with a phyloP score greater than 1.5: (**a**) Loads were estimated from protein-coding sites (**a**) or genome-wide (**b**). Names are color-coded for working horses (red), leisure horses (blue), and hotblood lines (green). Breeds considered of uncertain assignation are shown in grey. The red circle shows the F1 hybrid between a Przewalski and a domestic horse.

The Przewalski horse represents an excellent starting model for understanding the biological significance of mutational loads, owing to the population collapse experienced the 20th century, which led to their extinction in the wild in 1969. The now ~2100 animals living on the planet descend from a foundational captive stock of only 12–15 animals [30]. We first estimated individual loads within protein-coding regions, for comparison with previous work [5,20,31,32]. Averaging over 13 Przewalski horse genomes, and one Przewalski × Domestic F1 hybrid, we identified an average number of 1703.43 deleterious mutations out of 6,201,743 protein-coding sites. This corresponded to a mean load of approximately 3.698 × 10^−4^. As expected, most of the investigated breeds showed lower protein-coding loads than Przewalski horses, except for Shetland and Welsh ponies, as well as Marwari, Noriker, and Akhal Teke horses. Many of these breeds are presently represented by only one or two horse genomes; hence, we caution that the full range of possible load values present in these breeds remains to be explored. Other breeds such as Haflingers show highly variable loads, with some horses reaching values similar to those found in Przewalski horses. It is noteworthy that Haflingers and Norikers are draft breeds that are traditionally used as farm and pack animals. While only five major sire lines are described for Norikers, all modern purebred Haflingers can trace their ancestry back to one sire, Folie 249. Outbreeding was strictly prohibited in both breeds until recently, which limited founding stocks over multiple generations, leading to high load values (4.292 × 10^−4^ and 4.294 × 10^−4^, respectively; Figure 1A). Shetland ponies also ranked high, which was probably due to long periods of isolation and genetic drift in a small British island, and to the selective crossing policy developed since the creation of their breeding society in 1890 [33].

As they represent only a limited fraction of the genome, protein-coding regions might provide partial and potentially biased estimates for the mutational load. Thus, we expanded the calculation to also cover non-coding regions, including the 2 Kb flanking gene bodies, introns and intergenic regions. This increased the number of positions considered ~14-fold, representing approximately 67.6 million homozygous sites per genome. This also helped recover slightly and mildly constrained sites, which remained under-represented in protein-coding regions. This was so because protein-coding sites show increased evolutionary constraint relative to other regions, even at positions with phyloP scores greater than 1.5 (average phyloP scores, protein-coding = 2.316, 2 Kb upstream = 2.096, 2 Kb downstream = 2.097, intergenic = 1.964).

In general, the load estimates showed moderate correlation between the different regions considered (Spearman correlation; *ρ* < 0.586; *p*-value < 0.019), except for the protein-coding and 2 Kb upstream regions, where the correlation was non-significant (*p*-value = 0.949). We also found substantial differences on absolute scales, with loads within non-coding regions one order of magnitude greater (intergenic = 4.405 × 10^−3^, 2 Kb upstream = 4.034 × 10^−3^ and 2 Kb downstream = 3.972 × 10^−3^) than in protein-coding regions (3.476 × 10^−4^). Considering that most of the horse genome is non-coding, and that selection seems to be more efficient in purging strongly deleterious mutations within gene bodies, we concluded that, in horses, loads predominantly accumulate at non-coding sites, through multiple mutations of small fitness effect.

We next estimated genome-wide mutational loads, aiming at obtaining a fully representative set of positions, and potentially providing finer resolution for assessing the genomic consequences of different breed management practices. Results indeed revealed two major groups of breeds, each reflecting a major determinant of the current mutational burdens.

**Figure 2:**
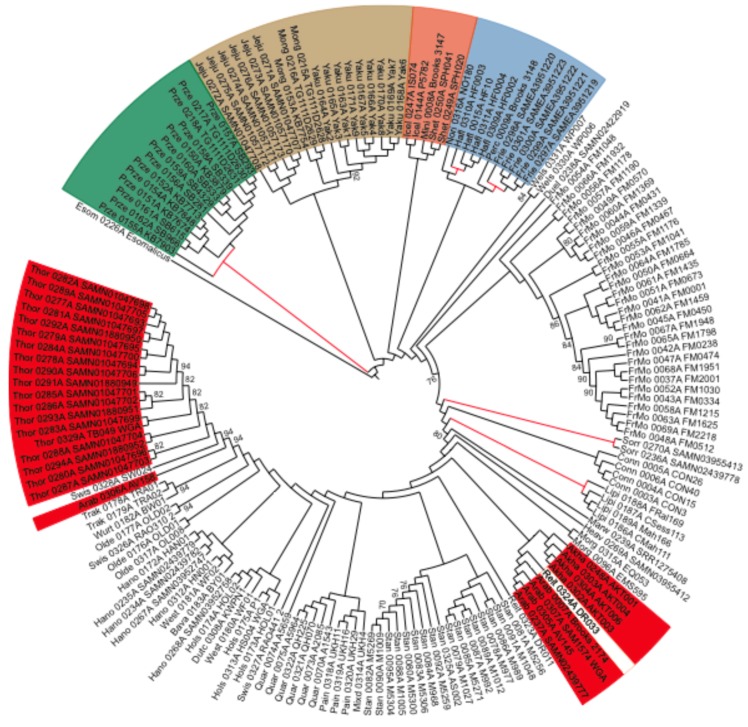
Neighbor-joining (NJ) cladogram depicting the horse phylogenetic relationships, mid-point rooted using the donkey as the outgroup. Color ranges highlight monophyletic lineages, including Przewalski horses (green), Mongolian-derived (brown) and Nordic (orange) breeds, as well as coldblooded (blue) and hotblooded (red) lines. Most of the non-colored taxa correspond to admixed warmblood lines, whose phylogenetic placement depends on the relative contribution of other influential breeds. Long internal branches are highlighted in red, as possibly reflecting episodes of elevated drift. Only bootstrap support values lower than 95% are displayed to avoid overloading the tree.

The top half of the load distribution is clearly dominated by traditional working breeds, including coldblood draft horses, as well as other farm and pack breeds (Figure 1B). We suggest that this owes to most working breeds being abandoned since the mechanization of locomotion. Their recent population collapse likely limited the efficacy of negative selection and inflated loads. Such is the case of South Korean Jeju horses, which collapsed after industrialization, and accumulated an excess of deleterious mutations (load = 4.301 × 10^−3^), and other draft and farm horses, such Lipizzans (4.297 × 10^−3^), Haflingers (4.292 × 10^−3^), and Norikers (4.294 × 10^−3^). Shetland ponies also show high genomic loads (4.310 × 10^−3^), which are even larger than those of the closely related Icelandic horses (4.294 × 10^−3^) (Figure 2). Both consisted originally of draft and farm animals, but have now been reconverted for leisure activities, and their census population size is limited. Likewise, the Marwari horse was endangered during the first half of the 20th century, until a series of conservation initiatives were started. The only Marwari representative analyzed in this study showed a genomic load of 4.312 × 10^−3^. This estimate was greater than that of Sorraia horses (4.283 × 10^−3^), which is a breed once thought to be extinct, until a relict population was discovered and recovered, albeit incorporating some farm specimens of uncertain genetic backgrounds [34]. It is noteworthy that the genomic burden was particularly pronounced for Friesian horses (4.428 × 10^−3^), the only breed exceeding the Przewalski mutational load genome-wide (4.310 × 10^−3^). However, two Friesian horses (SAMEA3951222 and SAMEA3951223) had much lower loads than the other three breed members. These two genomes were found to be more homozygous (37.1 versus 46.3 millions homozygous sites), despite being sequenced at comparable depths-of-coverage (~ 8–9 ×, Table S1). The bimodal pattern found for Friesian horses could be compatible with genetic purging [25], as further investigated and discussed below.

The bottom half of the full-genome load distribution is enriched in breeds that were originally engendered for sport and leisure. This mostly consisted of hotblood and warmblood lines, which show comparable loads despite being subject to different breeding strategies. On the one hand, hotblood lines such as Arabian (4.245 × 10^−3^) and Akhal-Teke (4.249 × 10^−3^) horses trace their origins deep in the past, possibly hundreds of years before the raise of modern breeding practices. The only exception pertains, precisely, to the more recently founded Thoroughbreds, for which studbook registration started in 1791, and loads are inflated (4.268 × 10^−3^). On the other hand, warmblood lines are more recent, but follow open stud guidelines that tolerate introgression from exogenous alleles, hence minimizing the deleterious effects of inbreeding (4.211–4.277 × 10^−3^, Figure 1B). Trakehners are worth a special mention, because unlike most warmblood horses, they are managed through nearly closed studbook practices, and expectedly show more elevated loads (4.281 × 10^−3^). The benefit of admixture is also evident in a series of breeds that incorporated ancient Arabian lines, such the Connemara, Welsh, Miniature, and Reit ponies, as well as the Percheron horse [35], which exhibit only moderated loads (Figure 1B). Finally, the Yakutian horse had the lowest loads (Figure 1B), with an average of 301,007 harmful alleles in the homozygous state, corresponding to a mean genomic load of 4.171 × 10^−3^. Their lowest genomic loads are in line with the incorporation of multiple reproductive stallions within the Yakutian genetic pool, as illustrated by their high Y-chromosomal diversity [36].

Overall, it appears that the incentive underlying the development of horse breeds, especially their main specialization as working or transport animals, has contributed to the mutational landscapes observed, with working breeds and coldblood lines showing greater genomic loads than hotblood and warmblood lines. To further assess this, we grouped horses according to the following categories (excluding breeds with uncertain assignation): (i) working horses (Jeju, Shetland, Icelandic, Haflinger, Percheron, Noriker, Friesian, Marwari, Sorraia, Lipizzan, and Franches Montagnes); (ii) hotblood (Akhal-Teke, Arabian, and Thoroughbreds); and (iii) warmblood horses (Bavarian, Oldenburger, Wurtemberg, Dutch, Hanoverian, Holsteiner, Morgan, American Paint, American Quarter, Standardbred, Trakehner, and Swiss Warmblood). We found significant statistical support for working horses carrying greater genomic loads than both hotblood (Wilcoxon test; *p*-value = 3.344 × 10^−3^) and the more admixed warmblood lines (Wilcoxon test; *p*-value = 6.256 × 10^−5^).

However, these groups showed no difference in their protein-coding loads (Figure 1A; Wilcoxon test; *p*-value > 0.1428). In addition to relying on a more limited number of positions, protein-coding loads represent regions evolving under stronger functional constraint, as reflected by their greater average phyloP scores. Computer simulations conducted by Fages et al. clearly indicated that, after a population collapse, load bursts are almost undetectable from strongly constrained sites as selection remains sufficiently effective, but can be detected at slightly deleterious variation (Figure S2 in [5]). Given that protein-coding loads seem far less sensitive to population collapses, it is thus not surprising that they fail to recover significant differences caused by the recent history of working, leisure, and hotblood lines.

### 3.2. Phylogenetic Reconstruction Supports Recent Population Decays in Working Breeds

To investigate population collapses, we built an NJ tree, which recovered strong bootstrap support for known phylogenetic relationships (Figure 2). In particular, Przewalski horses formed a sister group to all modern domesticates, with the F1 hybrid occupying the most basal position in this clade (Figure 2). Within domesticates, Mongolian, Jeju, and Yakutian horses split first, followed by a clade of Icelandic, Miniature, and Shetland ponies. Hotblooded Akhal-Tekke and Arabian horses clustered jointly, with a Reitpony specimen, which is a breed that is known to have been influenced by hotblood lines. However, Thoroughbreds formed their own cluster that was well separated from other hotblood lines. Coldblooded draft horses were also monophyletic, including Percherons, Friesians, Norikers, and Haflingers. Finally, warmblood horses were grouped per breed, but showed a more complex pattern of diversification, reflecting their more admixed nature and introgression from influential breeds, which were either cold or hotblooded.

We further scrutinized the length of internal branch lengths to potentially reveal past episodes of increased genetic drift. The longest internal branches led to Sorraia horses (7 × 10^−5^ substitutions per nearly-neutral site), Przewalski horses (6 × 10^−5^), Haflingers (5 × 10^−5^), Friesians (5 × 10^−5^), and Lipizzans (2.6 × 10^−5^) (Figure 2). The foundational branches of Icelandic (1.7 × 10^−5^), Shetland (1.7 × 10^−5^), and Jeju (1.5 × 10^−5^) ponies were slightly shorter, on par with those leading to Akhal-Teke (1.9 × 10^−5^) and Arabian (1.2 × 10^−5^) horses. These long internal branches echoed the mutational loads carried by working horses, suggesting independent demographic bottlenecks reducing the efficacy of negative selection and inflating their mutational loads. It is noteworthy that the branch length leading to all domesticates was only 1.7 × 10^−5^ substitutions per nearly-neutral site, despite encompassing the ~45,000 years of divergence with Przewalski horses [5]. This suggests that the genomic signature left by the domestication bottleneck was mild, and relative to that observed in some modern breeds. This mild bottleneck is consistent with the large mitochondrial diversity found in horses, which was interpreted as a pervasive restocking of wild mares during the initial spread of horse husbandry [37,38].

### 3.3. Deleterious Mutations Segregated at Low Frequencies Until the Last ~250 Years

**Figure 3:**
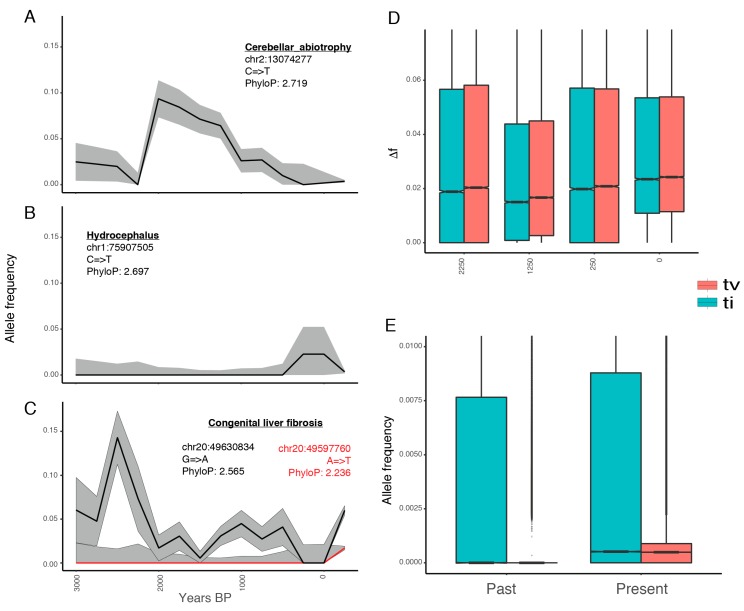
Temporal distribution of harmful variants: changes in frequency for alleles associated with (**a**) cerebellar abiotrophy, (**b**) hydrocephalus, and (**c**) congenital liver fibrosis; (**d**) Absolute differences in allele frequencies between time intervals (Δ). For example, point 2250 represents Δ from 3250 to 2250; (**e**) Frequency of deleterious mutations in ancient and modern horses, as estimated by ML. Ti and tv stand for nucleotide transitions and transversions, respectively.

We next aimed at reconstructing the past historical dynamics leading to present-day mutational loads. To achieve this, we first exploited genome-scale data from 153 ancient domestic horses and tracked the trajectories of all the deleterious alleles segregating in modern breeds over the last ~3500 years (*n* = 1,313,308). Figure 3A–C provide illustrative examples of the temporal trajectories of a selection of alleles known to be associated with diseases [39,40,41], including increasing and decreasing trends as well as cases where variation does not follow simple temporal changes. Overall, we detected that ~11.3% of the deleterious mutations were nearly fixed over time across all the periods, including in present-day domesticates (ML frequency ≥ 0.99). Thus, these harmful mutations spread prior to 3500 years ago, and probably prior to domestication. However, the vast majority of deleterious variants (~76.6%) remained nearly absent at all time periods (ML frequency < 0.01). This proportion increased to ~84.2% and ~86.9% when conditioning on more constrained positions (phyloP scores ≥ 2 and 2.5, respectively). This suggests that negative selection successfully maintained most of the deleterious variants at low frequencies during recent horse evolution.

We observed that the remaining fraction of deleterious variants (~12.0%, *n* = 158,448) followed a dynamic temporal trajectory, which is defined as detectable changes in frequency across successive time periods (Δ). To further quantify Δ, we conditioned on non-overlapping time bins of 1000 years, centered at 250, 1250, 2250, and 3250 years ago. This was done to ensure no redundancy across adjacent intervals, and hence to avoid underestimating Δ, since overlapping windows comprised of almost the same horses would provide nearly identical allele frequencies. We found that the most recent time interval tested, representing the last 250 years, experienced the largest shift in allele frequency (Figure 3D). Its median change over the 158,448 deleterious alleles was Δ = 0.02399, while it was 0.02048 or less for older time periods (Wilcoxon test; *p*-value < 2.2^−16^). These changes entailed increases or decreases in frequencies at equal proportions, except for the last 250 years, where 71% of deleterious variants became more common than in the previous time interval. This holds true when disregarding transitions, suggesting that the temporal trend was robust to the possible presence of post-mortem DNA misincorporations in the sequence data (although these were likely limited due to the treatment of most ancient DNA extracts with USER prior to DNA library preparation; Figure 3D).

As the results above supported those by Fages et al. [5], in which mutational loads were steady during millennia prior to the industrial revolution, we jointly considered all ancient specimens. This provided an approximately even number of 161 modern and 153 ancient horses to confidently estimate the frequencies of all the deleterious alleles (*n* = 1,313,308). On average, we detected that deleterious mutations are more common in modern horses, compared to their ancient relatives, by 0.3% according to transitions and by 0.8% to transversions (Figure 3E). Note that transitions are more spread than transversions also in modern horses, suggesting that their greater frequency is not due to post-mortem damage, but to sequencing biases and/or biological processes, such as CpG hypermutability and selection against transversions [42,43]. Taken together, these findings confirm that current deleterious mutations segregated in the past, but that it was only recently that they significantly rose in frequency.

### 3.4. Inbreeding Depression and Genetic Purging Shaped Loads in Modern Domesticates

Understanding the evolutionary mechanisms that forged current mutational loads is paramount to identifying (un)desirable breeding practices and designing more sustainable strategies. Given the recent time-scale delimited within the last 250 years, inbreeding depression represents the most likely mechanism underlying the recent increase of mutational loads in the horse genome [44,45]. Inbreeding depression is caused by recessive mutations that are phenotypically hidden in the heterozygous state, until they become effective once located within the runs of homozygosity (ROHs) introduced by inbreeding. Assuming that inbreeding depression was the main driver of the mutational load patterns observed in this study, we should expect that: (1) recessive mutations segregated in the ancestral population, and (2) negative selection was not sufficiently strong to remove recessive mutations exposed within ROHs [25].

**Figure 4:**
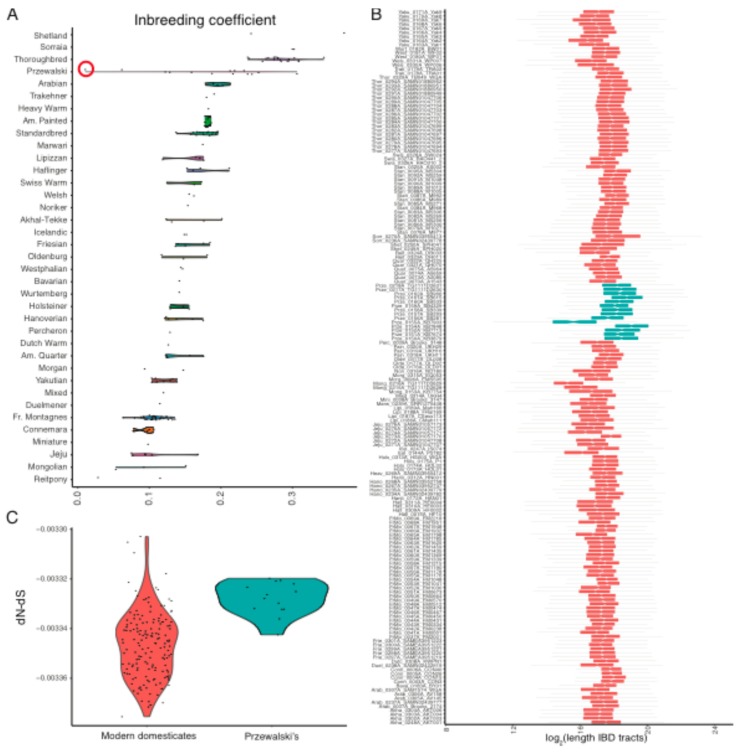
Inbreeding depression and genetic purging: (**a**) Inbreeding per breed; (**b**) Box plot summarizing the length of ROHs in a given individual horse. The ROH length is represented in log_2_ scale to avoid excessive distortion caused by outliers. In this scale, a value of 18 corresponds to 2^18^ = 262 Kb, while a value of 24 corresponds to 2^24^ = 16 Mb; (**c**) Non-synonymous (dN)˗synonymous (dS) substitution rates, for Przewalski horses and modern domesticates. More negative dN˗dS values reflect more efficient selection. The specimen highlighted with a red circle corresponds to sample KB7903 in Der Sarkissian et al. 2015, and represents an F1 hybrid between a Przewalski horse and a domestic horse.

The first assertion was proved in the section above. In order to test the second, we characterized inbreeding and identified ROHs in each individual modern horse genome using ngsF-HMM [29]. Our inbreeding estimates replicated previous work for the 14 Przewalski horses [46], showing an average inbreeding coefficient of *F* = 18.5%, corresponding to ~18.5% of the genome being identical-by-descendant (IBD) (Figure 4A). The Przewalski x Domestic F1 hybrid analyzed (KB7903) showed no inbreeding, and also had the lowest mutational load in the group (Figure 1B). This suggests that the inbreeding estimates that were recovered are genuine. We found that inbreeding coefficients and mutational loads were strongly correlated in Przewalski horses (Spearman correlation; *ρ* = 0.903; *p*-value = 9.740 × 10^−6^), which was as expected if negative selection was not sufficiently strong to eliminate the deleterious variants exposed in ROHs.

Modern domesticates returned slightly lower inbreeding coefficients (on average, *F =* 15.9%). This estimate is greater than the 8.8% recently estimated across nine breeds, based on 65,157 SNPs only [47], suggesting strong ascertainment bias within this SNP set. Ranking per breed revealed that Shetland, Sorraia, and Thoroughbreds were extremely inbred, with values approaching and even exceeding *F* = 30% (Figure 4A). Their longest IBD tracts spanned 20 Mb, 10 Mb, and 11 Mb, respectively (Figure 4B). Interestingly, and in contrast to Przewalski horses, inbreeding did not necessarily entail increased loads, as the correlation was weaker, albeit significant (*ρ* = 0.222; *p*-value = 4.708 × 10^−3^). Limiting the calculations to those 53 domesticates sequenced above 15 × strengthened the correlation coefficient (*ρ* = 0.4754); however, it remained inferior to that inferred for Przewalski horses. A similar trend was found conditioning load estimates on protein-coding sites. This suggests that additional mechanisms, beyond inbreeding depression, have contributed to shape the mutational load present in modern breeds.

As genetic purging involves strong selection against recessive mutations exposed within ROHs, we propose that this mechanism could have reduced the mutational loads in the most inbred horses. This mechanism may have been inefficient in Przewalski horses due to the extremely limited reproductive stock available for the conservation program and/or to the favorable environmental conditions offered in captivity and reintroduction reserves. To further validate whether selection was stronger in modern domesticates than in Przewalski horses, we quantified the difference between non-synonymous and synonymous mutation rates, dN–dS, in each individual genome (see methods). All the horses had negative dN–dS values, as expected under negative selection (Figure 4C). Yet, Przewalski horses appeared at the higher end of the dN–dS distribution (mean dN–dS = −3.328 × 10^−3^), confirming more relaxed negative selection in this lineage relative to domestic horses. Amongst modern domesticates, the highly isolated Shetland ponies represent the only breed showing lower negative selection than Przewalski horses (−3.306 × 10^−3^). Interestingly, Thoroughbreds were found to be at the tail of the dN–dS distribution (−3.366 × 10^−3^). The correlation between load and dN–dS, which was calculated across the 19 Thoroughbreds investigated, was non-significant (*ρ* = 0.178; *p*-value = 0.467), supporting ongoing genetic purging in this breed.

## 4. Discussion

Recent work from Fages et al. revealed that the last few centuries have been accompanied by a ~16% drop in the horse heterozygosity genome-wide, and a ~4% raise in the mutational load within protein-coding regions [5]. However, the underlying drivers of these shifts remained unclear. To address this gap, we carried out an extensive characterization of mutational loads in horses, leveraging previously published genome data from a total of 175 modern horses spanning 37 breeds and/or populations, and 153 ancient horses. We expanded the calculation of mutational loads outside protein-coding regions, which enhanced both resolution and accuracy.

Our findings support inbreeding depression as the main mechanism driving the load burst in domesticates and Przewalski horses. It is important to keep in mind that the last ~250 years cannot generate sufficient amounts of de novo variants to explain the observed increment in load, given the low mutation rate inferred for horses [48]. Therefore, the excess of mutational load was almost entirely driven by standing variation; it was also likely located in non-coding regions, and associated with slightly deleterious and recessive inheritance (i.e., dominant deleterious mutations are less frequent, because they are phenotypically exposed to negative selection at the heterozygous state, and thus efficiently eliminated). We indeed confirm that deleterious variation segregated in the past, at very low frequencies, until rising recently. Nevertheless, their increments of only ~0.3% (transitions) and 0.8% (transversions) is lower than the 4% raise in protein-coding loads [5]. Hence, the load burst cannot be only explained by a higher frequency of deleterious mutations, but requires that they increasingly became exposed at the homozygous state, owing to inbreeding. The significant correlation between inbreeding and the mutational load identified here further corroborates the role of inbreeding in causing a fitness depression.

Inbreeding was caused by two main historical shifts. The ubiquity of steam and combustion vehicles relegated breeds traditionally used for farming and transport to almost oblivion, resulting in fast population collapses (and even extinction in some cases) [49]. Although inbreeding exposes recessive deleterious variants to negative selection within ROHs, the reduced effective sizes considerably limited the efficacy of negative selection. For example, this increased mutational loads in Sorraia, Haflingers, Norikers, and especially Friesian horses, which show larger mutational loads than the endangered Przewalski horses (Figure 1B). One exception to this pattern pertains to one single Percheron horse present in our genome dataset. This breed consisted of large, draft horses, and is known to have been extremely influenced by Arabian bloodlines while being founded in France, before becoming extremely popular worldwide, especially in the U.S., in the 19th century [50]. As a result, its geographic range was exceptionally large for a draft breed, which may have helped preserve sufficient genetic diversity, until conservation programs started in the 1960s.

Conservation programs often rely on closed stud practices to maintain un-admixed populations that are better adapted to native environments. For endangered breeds, this implies that foals can only be registered if descending from purebred studbook-registered parents. Similar rules govern selection programs to improve specific breeds, such as Thoroughbreds, for which a studbook was established well before Darwin formalized the concept of evolution through natural selection in 1859 [51]. Closed studs, and the preferential reproduction of influential stallions, increased inbreeding and the probability of exposing deleterious alleles in the homozygous state. In extremely large studs such as Thoroughbreds, which are intensively selected for performance, this may have provided sufficient strength to purge deleterious mutations. However, in other breeds, which are either less intensively selected or restricted to extremely low effective sizes, mutational loads could spread.

The abandonment of working breeds and the emergence of closed studbooks clearly post-date the onset of domestication by at least five millennia [32,52,53]. This has deep implications for the cost-of-domestication hypothesis [54], which posits that there was an increase in mutational load due to the repeated bottlenecks presumably experienced during the early domestication stages [31,55,56,57]. In agreement with previous work in horses [20] and crops [58], we find that this is not necessarily the case, and highlight that two forces with opposing effects could have also contributed to shape mutational loads. On the one hand, the impact of recent events seems unprecedented in the horse evolutionary history, and appears to have eroded the horse genome more than the domestication bottleneck itself (Figure 2 and Figure 3). On the other hand, restocking from the wild and cross-breeding during hundreds of generations could have counteracted the deleterious consequences of early population declines, e.g., through heterosis, which is a phenomenon involving greater vigor and fertility in hybrids than in their parental inbreed stocks [13].

A number of mathematical models for inbreeding depression, heterosis, and genetic purging [14,25,59,60] have predicted that populations with reproductive stocks that are comparable to what are found in many domestic breeds undergo a strong risk of extinction. For example, Caballero et al. recently used simulations to estimate that populations limited to approximately 70 reproductive individuals are under substantial risk of extinction, as defined by a >10% reduction in viability after 50 generations of evolution [61]. Note that a breeding stock of *N_m_* = 20 stallions and *N_f_* = 5000 mares corresponds to a population size of only *N_e_* = 4*N_m_N_f_/(N_m_+N_f_)* ≈ 80 individuals. In line with this, and according to the Domestic Animal Diversity Information System (DAD-IS; accessed 19 July 2019 from the FAO website [49]), more than 200 horse breeds would thus be endangered or at the brink of extinction, while 88 are already extinct.

Encouragingly, the relation between inbreeding and mutational loads was found to be impacted by genetic purging in modern domesticates (Figure 4). Thus, genetic purging adds an extra layer of complexity to the interplay of forces that forged genomic loads, not only helping to improve fitness, but also to optimize traits that are paramount to the equine industry. For example, in Thoroughbreds, genetic purging has been associated with improved racing performance [62]. This means that breeders can leverage genomic information to design mating strategies favoring the purging of deleterious mutations from the breeding stock, improving animal welfare and mitigating extinction risks.

## Supplementary Materials

The following are available online at https://www.mdpi.com/2073-4425/10/9/649/s1, Table S1: Metadata for 175 modern horses investigated in this study. Table S2: Metadata for 153 ancient horses investigated in this study.

## References

[1] Anthony D.W. (2010). The Horse, the Wheel, and Language: How Bronze-Age Riders from the Eurasian Steppes Shaped the Modern World.

[2] Kelekna P. (2009). The Horse in Human History.

[3] Equine Industry Statistics Overview l Equine Business Association. https://www.equinebusinessassociation.com/equine-industry-statistics/.

[4] Librado P., Fages A., Gaunitz C., Leonardi M., Wagner S., Khan N., Hanghøj K., Alquraishi S.A., Alfarhan A.H., Al-Rasheid K.A. (2016). The Evolutionary Origin and Genetic Makeup of Domestic Horses. Genetics.

[5] Fages A., Hanghøj K., Khan N., Gaunitz C., Seguin-Orlando A., Leonardi M., McCrory Constantz C., Gamba C., Al-Rasheid K.A.S., Albizuri S. (2019). Tracking Five Millennia of Horse Management with Extensive Ancient Genome Time Series. Cell.

[6] Wallner B., Vogl C., Shukla P., Burgstaller J.P., Druml T., Brem G. (2013). Identification of Genetic Variation on the Horse Y Chromosome and the Tracing of Male Founder Lineages in Modern Breeds. PLoS ONE.

[7] Felkel S., Vogl C., Rigler D., Dobretsberger V., Chowdhary B.P., Distl O., Fries R., Jagannathan V., Janečka J.E., Leeb T. (2019). The horse Y chromosome as an informative marker for tracing sire lines. Sci. Rep..

[8] Bosse M., Megens H., Derks M.F.L., de Cara Á.M.R., Groenen M.A.M. (2018). Deleterious alleles in the context of domestication, inbreeding, and selection. Evol. Appl..

[9] Szpiech Z.A., Xu J., Pemberton T.J., Peng W., Zöllner S., Rosenberg N.A., Li J.Z. (2013). Long Runs of Homozygosity Are Enriched for Deleterious Variation. Am. J. Hum. Genet..

[10] Nicholas F.W. (2003). Online Mendelian Inheritance in Animals (OMIA): A comparative knowledgebase of genetic disorders and other familial traits in non-laboratory animals. Nucleic Acids Res..

[11] Korte A., Farlow A. (2013). The advantages and limitations of trait analysis with GWAS: A review. Plant. Methods.

[12] Charlesworth B. (2009). Effective population size and patterns of molecular evolution and variation. Nat. Rev. Genet..

[13] Charlesworth D., Willis J.H. (2009). The genetics of inbreeding depression. Nat. Rev. Genet..

[14] Lynch M., Conery J., Burger R. (1995). Mutation Accumulation and the Extinction of Small Populations. Am. Nat..

[15] Understanding and Predicting the Fitness Decline of Shrunk Populations: Inbreeding, Purging, Mutation, and Standard Selection|Genetics. https://www.genetics.org/content/190/4/1461.long.

[16] Schubert M., Ermini L., Der Sarkissian C., Jónsson H., Ginolhac A., Schaefer R., Martin M.D., Fernández R., Kircher M., McCue M. (2014). Characterization of ancient and modern genomes by SNP detection and phylogenomic and metagenomic analysis using PALEOMIX. Nat. Protoc..

[17] Siepel A., Pollard K.S., Haussler D., Apostolico A., Guerra C., Istrail S., Pevzner P.A., Waterman M. (2006). New Methods for Detecting Lineage-Specific Selection. Proceedings of the Research in Computational Molecular Biology.

[18] Pollard K.S., Hubisz M.J., Rosenbloom K.R., Siepel A. (2010). Detection of nonneutral substitution rates on mammalian phylogenies. Genome Res..

[19] Wade C.M., Giulotto E., Sigurdsson S., Zoli M., Gnerre S., Imsland F., Lear T.L., Adelson D.L., Bailey E., Bellone R.R. (2009). Genome sequence, comparative analysis, and population genetics of the domestic horse. Science.

[20] Librado P., Gamba C., Gaunitz C., Sarkissian C.D., Pruvost M., Albrechtsen A., Fages A., Khan N., Schubert M., Jagannathan V. (2017). Ancient genomic changes associated with domestication of the horse. Science.

[21] Korneliussen T.S., Albrechtsen A., Nielsen R. (2014). ANGSD: Analysis of Next Generation Sequencing Data. BMC Bioinform..

[22] Dabney J., Meyer M., Pääbo S. (2013). Ancient DNA Damage. Cold Spring Harb. Perspect. Biol..

[23] Purcell S., Neale B., Todd-Brown K., Thomas L., Ferreira M.A.R., Bender D., Maller J., Sklar P., de Bakker P.I.W., Daly M.J. (2007). PLINK: A tool set for whole-genome association and population-based linkage analyses. Am. J. Hum. Genet..

[24] Lefort V., Desper R., Gascuel O. (2015). FastME 2.0: A Comprehensive, Accurate, and Fast Distance-Based Phylogeny Inference Program. Mol. Biol. Evol..

[25] Hedrick P.W., Garcia-Dorado A. (2016). Understanding Inbreeding Depression, Purging, and Genetic Rescue. Trends Ecol. Evol..

[26] Hill W.G., Robertson A. (1968). Linkage disequilibrium in finite populations. Theor. Appl. Genet..

[27] Fox E.A., Wright A.E., Fumagalli M., Vieira F.G. (2019). ngsLD: Evaluating linkage disequilibrium using genotype likelihoods. Bioinformatics.

[28] Van Dongen S. (2008). Graph Clustering Via a Discrete Uncoupling Process. SIAM J. Matrix Anal. Appl..

[29] Vieira F.G., Albrechtsen A., Nielsen R. (2016). Estimating IBD tracts from low coverage NGS data. Bioinformatics.

[30] The IUCN Red List of Threatened Species. https://www.iucnredlist.org/en.

[31] Schubert M., Jónsson H., Chang D., Der Sarkissian C., Ermini L., Ginolhac A., Albrechtsen A., Dupanloup I., Foucal A., Petersen B. (2014). Prehistoric genomes reveal the genetic foundation and cost of horse domestication. Proc. Natl. Acad. Sci. USA.

[32] Gaunitz C., Fages A., Hanghøj K., Albrechtsen A., Khan N., Schubert M., Seguin-Orlando A., Owens I.J., Felkel S., Bignon-Lau O. (2018). Ancient genomes revisit the ancestry of domestic and Przewalski’s horses. Science.

[33] Shetland Ponies, about Shetland Ponies—The Breed and Stud-Book. http://www.shetlandponystudbooksociety.co.uk/about-the-breed.

[34] Luís C., Cothran E.G., Oom M.d.M. (2007). Inbreeding and Genetic Structure in the Endangered Sorraia Horse Breed: Implications for its Conservation and Management. J. Hered..

[35] Horses—Breeds of Livestock, Department of Animal Science. http://afs.okstate.edu/breeds/horses/horses-w.html#r.

[36] Librado P., Sarkissian C.D., Ermini L., Schubert M., Jónsson H., Albrechtsen A., Fumagalli M., Yang M.A., Gamba C., Seguin-Orlando A. (2015). Tracking the origins of Yakutian horses and the genetic basis for their fast adaptation to subarctic environments. Proc. Natl. Acad. Sci. USA.

[37] Achilli A., Olivieri A., Soares P., Lancioni H., Hooshiar Kashani B., Perego U.A., Nergadze S.G., Carossa V., Santagostino M., Capomaccio S. (2012). Mitochondrial genomes from modern horses reveal the major haplogroups that underwent domestication. Proc. Natl. Acad. Sci. USA.

[38] Jansen T., Forster P., Levine M.A., Oelke H., Hurles M., Renfrew C., Weber J., Olek K. (2002). Mitochondrial DNA and the origins of the domestic horse. Proc. Natl. Acad. Sci. USA.

[39] Drögemüller M., Jagannathan V., Welle M.M., Graubner C., Straub R., Gerber V., Burger D., Signer-Hasler H., Poncet P.-A., Klopfenstein S. (2014). Congenital Hepatic Fibrosis in the Franches-Montagnes Horse Is Associated with the Polycystic Kidney and Hepatic Disease 1 (PKHD1) Gene. PLoS ONE.

[40] Brault L.S., Cooper C.A., Famula T.R., Murray J.D., Penedo M.C.T. (2011). Mapping of equine cerebellar abiotrophy to ECA2 and identification of a potential causative mutation affecting expression of MUTYH. Genomics.

[41] Ducro B.J., Schurink A., Bastiaansen J.W.M., Boegheim I.J.M., van Steenbeek F.G., Vos-Loohuis M., Nijman I.J., Monroe G.R., Hellinga I., Dibbits B.W. (2015). A nonsense mutation in B3GALNT2 is concordant with hydrocephalus in Friesian horses. BMC Genomics.

[42] Zhang J. (2000). Rates of conservative and radical nonsynonymous nucleotide substitutions in mammalian nuclear genes. J. Mol. Evol..

[43] Lyons D.M., Lauring A.S. (2017). Evidence for the Selective Basis of Transition-to-Transversion Substitution Bias in Two RNA Viruses. Mol. Biol. Evol..

[44] O’Grady J.J., Brook B.W., Reed D.H., Ballou J.D., Tonkyn D.W., Frankham R. (2006). Realistic levels of inbreeding depression strongly affect extinction risk in wild populations. Biol. Conserv..

[45] Ebert D., Haag C., Kirkpatrick M., Riek M., Hottinger J.W., Pajunen V.I. (2002). A selective advantage to immigrant genes in a Daphnia metapopulation. Science.

[46] Der Sarkissian C., Ermini L., Schubert M., Yang M.A., Librado P., Fumagalli M., Jónsson H., Bar-Gal G.K., Albrechtsen A., Vieira F.G. (2015). Evolutionary Genomics and Conservation of the Endangered Przewalski’s Horse. Curr. Biol..

[47] Genes|Free Full-Text|The Genomic Makeup of Nine Horse Populations Sampled in the Netherlands. https://www.mdpi.com/2073-4425/10/6/480.

[48] Orlando L., Ginolhac A., Zhang G., Froese D., Albrechtsen A., Stiller M., Schubert M., Cappellini E., Petersen B., Moltke I. (2013). Recalibrating Equus evolution using the genome sequence of an early Middle Pleistocene horse. Nature.

[49] Data export|Domestic Animal Diversity Information System (DAD-IS)|Food and Agriculture Organization of the United Nations. http://www.fao.org/dad-is/dataexport/en/.

[50] Mischka J. (1991). The Percheron Horse in America.

[51] Montgomery E.S. (1972). The Thoroughbred.

[52] Outram A.K., Stear N.A., Bendrey R., Olsen S., Kasparov A., Zaibert V., Thorpe N., Evershed R.P. (2009). The Earliest Horse Harnessing and Milking. Science.

[53] Ludwig A., Pruvost M., Reissmann M., Benecke N., Brockmann G.A., Castaños P., Cieslak M., Lippold S., Llorente L., Malaspinas A.-S. (2009). Coat Color Variation at the Beginning of Horse Domestication. Science.

[54] Lu J., Tang T., Tang H., Huang J., Shi S., Wu C.-I. (2006). The accumulation of deleterious mutations in rice genomes: A hypothesis on the cost of domestication. Trends Genet..

[55] Marsden C.D., Vecchyo D.O.-D., O’Brien D.P., Taylor J.F., Ramirez O., Vilà C., Marques-Bonet T., Schnabel R.D., Wayne R.K., Lohmueller K.E. (2016). Bottlenecks and selective sweeps during domestication have increased deleterious genetic variation in dogs. Proc. Natl. Acad. Sci. USA.

[56] Koenig D., Jiménez-Gómez J.M., Kimura S., Fulop D., Chitwood D.H., Headland L.R., Kumar R., Covington M.F., Devisetty U.K., Tat A.V. (2013). Comparative transcriptomics reveals patterns of selection in domesticated and wild tomato. Proc. Natl. Acad. Sci. USA.

[57] Moyers B.T., Morrell P.L., McKay J.K. (2018). Genetic Costs of Domestication and Improvement. J. Hered..

[58] Allaby R.G., Ware R.L., Kistler L. (2018). A re-evaluation of the domestication bottleneck from archaeogenomic evidence. Evol. Appl..

[59] Wang J., Hill W.G., Charlesworth D., Charlesworth B. (1999). Dynamics of inbreeding depression due to deleterious mutations in small populations: Mutation parameters and inbreeding rate. Genet. Res..

[60] Charlesworth B. (2018). Mutational load, inbreeding depression and heterosis in subdivided populations. Mol. Ecol..

[61] Caballero A., Bravo I., Wang J. (2017). Inbreeding load and purging: Implications for the short-term survival and the conservation management of small populations. Heredity.

[62] Todd E.T., Ho S.Y.W., Thomson P.C., Ang R.A., Velie B.D., Hamilton N.A. (2018). Founder-specific inbreeding depression affects racing performance in Thoroughbred horses. Sci. Rep..

